# Tear proteome profile in eyes with keratoconus after intracorneal ring segment implantation or corneal crosslinking

**DOI:** 10.3389/fmed.2022.944504

**Published:** 2022-09-20

**Authors:** Nahia Goñi, Itziar Martínez-Soroa, Oliver Ibarrondo, Mikel Azkargorta, Felix Elortza, David J. Galarreta, Arantxa Acera

**Affiliations:** ^1^Department of Ophthalmology, Hospital Universitario Donostia, San Sebastian, Spain; ^2^Department of Ophthalmology, University of the Basque Country UPV/EHU, Leioa, Spain; ^3^RS-Statistics, Arrasate-Mondragón, Spain; ^4^Proteomics Platform, CIC bioGUNE, Basque Research and Technology Alliance (BRTA), CIBERehd, Derio, Spain; ^5^Department of Ophthalmology, Hospital Clínico Universitario de Valladolid, Valladolid, Spain; ^6^Department of Cell Biology and Histology, Experimental Ophthalmo-Biology Group (GOBE: www.ehu.eus/gobe), University of the Basque Country UPV/EHU, Leioa, Spain; ^7^IKERBASQUE, Basque Foundation for Science, Bilbao, Spain

**Keywords:** keratoconus, tear film, intracorneal ring segment, crosslinking, biomarker

## Abstract

**Purpose:**

Keratoconus (KC) is a corneal ectasia characterized by structural changes, resulting in progressive thinning and biomechanical weakening that can lead to worsening visual acuity due to irregular astigmatism. Corneal collagen Crosslinking (CXL) and Intracorneal Ring Segment (ICRS) are widely used treatments in KC disease, but the alterations they cause in biomechanical mediators are still poorly understood. The aim of this study was to analyze the tear proteome profile before and after treatments to identify biomarkers altered by surgery.

**Materials and methods:**

An observational, prospective, case-control pilot study was conducted, analyzing tear samples from KC patients by nano-liquid chromatography-mass spectrometry (nLC-MS/MS). Data are available *via* ProteomeXchange with identifier PXD035655. Patients with KC who underwent ICRS surgery (*n* = 4), CXL (*n* = 4), and healthy subjects (Ctrl, *n* = 4) were included in this study. Clinical parameters were measured and tear samples were collected before and 18 months after surgery. Proteins with ≥2 expression change and *p*-value < 0.05 between groups and times were selected to study their role in post-operative corneal changes.

**Results:**

These analyses led to the identification of 447 tear proteins, some of which were dysregulated in KC patients. In comparisons between the two surgical groups and Ctrls, the biological processes that were altered in KC patients at baseline were those that were dysregulated as a consequence of the disease and not of the surgical intervention. Among the biological processes seen to be altered were: immune responses, cytoskeleton components, protein synthesis and metabolic reactions. When comparing the two treatment groups (ICRS and CXL), the process related to cytoskeleton components was the most altered, probably due to corneal thinning which was more pronounced in patients undergoing CXL.

**Conclusion:**

The changes observed in tears after 18 months post-operatively could be due to the treatments performed and the pathology. Among the deregulated proteins detected, A-kinase anchor protein 13 (AKAP-13) deserves special attention for its involvement in corneal thinning, and for its strong overexpression in the tears of patients with more active KC and faster disease progression. However, it should be kept in mind that this is a pilot study conducted in a small number of patients.

## Introduction

Keratoconus (KC) is the most common primary corneal ectasia with an estimated incidence of 1 in 2,000 among the general population (1). It is a bilateral and asymmetric pathology that is characterized by a progressive thinning and protrusion of the cornea, predominantly in its inferotemporal or central region. It usually commences at puberty and progresses until the third or fourth decade of life, at which point it does not progress further (2). Less frequently it may initiate earlier in life, following a more aggressive and rapid progression (2–4). There is some variability in the presentation and evolution of this condition, although changes in ocular refraction provoked by the development of irregular astigmatism and the consequent loss of visual acuity (VA) are characteristic features of KC (5–7). The macroscopic and microscopic alterations that can be observed depend on the stage of KC and they include central or paracentral stromal thinning, the protrusion of the cornea as a cone, Fleischer rings, Vogt’s striae, prominent corneal nerves, Munson’s and Rizzuti’s signs, sub-epithelial opacity, and stromal scarring (1).

Despite the studies carried out to date the precise details of the physiopathology of KC remain unknown. It appears to be of multifactorial origin, combining genetic and environmental factors (8–13). Among the factors identified are atopy, chronic eye rubbing or exposure to ultraviolet (UV) light, although there is no clear consensus as to the overall importance of each of these in the physiopathology of the disease (12). Over and above the risk factors that favor the development of this disease are the internal events that give rise to the structural changes associated with this pathology. Proteomics studies of the distinct corneal layers, the tear film and the aqueous humor have provided fundamental information in order to understand the processes that take place during the development of KC (14). Accordingly, inflammation, oxidative stress, enzyme deregulation and cellular hypersensitivity are the pathophysiological events most often described (14–20). Over the years, several studies have been conducted to characterize the human tear proteome using different proteomic approaches. Based on the most recent literature, the tear proteome is estimated to be around 1500 proteins (21) of which 10% are extracellular. However, although the number of extracellular proteins is not so high, it is instead in terms of their relative concentration and this is due not only to the contribution of the lacrimal gland, but also to the contribution of both corneal and conjunctival epithelial cells. Studies of the tear proteome have identified different patterns of proteins associated with specific pathologic conditions (22, 23). These studies illustrated the usefulness and importance of tear component analysis as a source of insight into the pathologic mechanisms involved in ocular surface disorders. Studies on KC have demonstrated the presence of alterations in the corneal epithelium (24) and stroma (25), but complementary and useful information can also be obtained by biochemical analysis of tears, which are relatively easy to obtain. Tear film stability is a prerequisite for proper optical and metabolic functioning of the eye. It is important to know and understand the terms related to tear stability, the phenomena involved in the different theories proposed, and the techniques to evaluate it in order to make a more accurate diagnosis, leading to effective treatments.

The optimal treatment of KC includes personalized approaches that take into account an evaluation of the patient’s visual demands, the degree of KC, its progression or stability, age, and tolerance to visual correction with glasses or contact lenses. Conservative treatment with glasses or contact lenses is reserved for those patients with stable KC or after a surgical procedure, such as cross-linking (CXL), intracorneal ring segment (ICRS) implantation or keratoplasty. The therapeutic approach in young patients with progressive KC has been modified by the introduction of CXL, a surgical procedure that halts its progression. This is a procedure particularly recommended for young patients (especially under 25 years of age) with progressive KC, pachymetry above 400 microns, a clear cornea and keratometry values below 60 diopters (D). By contrast, in patients with stable KC, visual affectation and poor adaptation to optical correction, a minimal pachymetry of 400 microns, a clear central cornea and keratometry values below 60D the recommended procedure is ICRS. Deep Anterior Lamellar Keratoplasty (DALK) and Penetrating Keratoplasty (PK) are only considered in cases that do not meet these indications (26–28).

Crosslinking is a chemical reaction that involves the covalent binding of two or more molecules, changing their physicochemical properties. This is achieved by stimulating crosslinking molecules with physical agents (UV light, heat, pressure), chemical catalysis, or a combination of both (29). The efficacy of CXL with UVA radiation and riboflavin was described in patients with KC, producing a 328.9% increase in the rigidity of the cornea that would explain the positive benefits of CXL in stabilizing KC (30). This efficacy of this approach was later confirmed in clinical studies (31–35). Subsequently, the conventional Dresden protocol was proposed that involved the desepithelialization of the cornea by applying a riboflavin solution (0.1%) for 30 min, followed by irradiation at a wavelength of 370 nm and with a potency of 3 mW/cm^2^ (36). Subsequently, distinct variations of this technique were published using the same surgical technique to limit the adverse effects of conventional CXL. Accelerated CXL, for example, reduces surgical time by applying a higher irradiation dose for a shorter period of time (37).

The implantation of ICRS (33), circular segments of poly(methyl methacrylate) flattens the cornea and recovers its curvature, reducing any astigmatism and representing an alternative to improve the patients vision when astigmatism is strong and/or they tolerate contact lenses poorly (38, 39). These implants act as passive spacers and when placed on the cornea, they provoke the local separation of the corneal layers, which results in a shortening of the anterior corneal curvature and a flattening of the central cornea (29). When only one segment is used, flattening only occurs in that region and there is an increase in curvature in the opposite direction of the ring. Moreover, the thicker the segment, the greater the flattening produced (40). The ICRS can be implanted manually or using a femtosecond laser. Both these procedures have similar clinical results but the use of femtosecond laser is associated with fewer intraoperative complications, and greater precision and predictability (41–43). The distribution of the stress on shortening the layers and changing the shape of the cornea will alter the cycle and the pattern of progressive decompensation, permitting the cornea to adopt a more regular shape over time. Likewise, the natural evolution of the disease toward a stable state could be a determinant in the post-operative progression. However, at present it is unclear what the impact of this treatment might be on the underlying processes (44). The tear is a complex biological fluid that contains mucin, proteins/peptides, electrolytes, lipids, and metabolites.

Despite its relatively small volumes, the tear composition reflects the physiological status of the eye and its underlying systems, and it can provide information regarding ophthalmological and systemic pathologies (21). In fact, studying the variations in the composition of the tear is a good approach to discover biomarkers. As such, proteomic studies of the tear could explain the changes that occur in the cornea and at the eye surface after CXL and ICRS implantation. The objective of this study was to describe the proteomic changes induced in the tear of patients with KC after performing these two surgical procedures.

## Materials and methods

### Study cohort

An observational, prospective, interventional case-control pilot study was designed, in which 8 patients with KC and 4 healthy subjects (Ctrl) were included. Patients with KC underwent 2 types of surgery and as such, they were divided into two groups. Group 1 underwent Ferrara ICRS implantation surgery and group 2 underwent corneal accelerated CXL. Group 3 consisted of the 4 Ctrl. This research was carried out by qualified medical personnel after receiving approval from the Ethics Committee at the Hospital Universitario Donostia (Code 2015120). The study was carried out in strict accordance with the principles of the Helsinki Declaration on Biomedical Research Involving Human Subjects. Prior to sample collection, signed informed consent was obtained from all subjects or their legal representatives (in the case of patients under 18 years of age) after the nature and possible consequences of the study were explained.

Patients were recruited consecutively at the Ophthalmology Service of the Hospital Universitario Donostia (San Sebastian, Gipuzkoa, Spain) during outpatient consultations, between January 2018 and February 2019. The follow-up of the patients continued until February 2020. The inclusion criteria applied to the group of patients was the prior diagnosis of KC [mild, moderate or severe (6)], age between 14 and 45 years, and the need to perform a surgical procedure to treat their KC (27). The diagnostic criteria used to confirm KC were based on the topographic criteria of Rabinowitz (1) and the Belin-Ambrosi o algorithm incorporated into the Pentacam^®^ Software (Pentacam HR; Oculus Optikgerate GmbH, Wetzlar, Germany) regarding corneal thickness (Belin/Ampbrosio Enhanced Ectasia Display). The exclusion criteria included any eye surgery carried out prior to recruitment, systemic corticoid or anti-depressive medication, chronic eye medication except for artificial tears or topical anti-histamines, a mean keratometry above 60D or pachymetry below 400 microns. Contact lens wearers were asked to desist from wearing contact lenses for 15 days prior to any testing to avoid any possible interference in the interpretation of results and all patients were asked to avoid scratching their eyes prior to surgeries. Preliminary eye tests and sample collection was performed on the same day. Two visits were performed, a pre-surgical baseline visit and a post-surgical visit 18 months later.

The patients that displayed a reduction in at least 1 line of vision, an increase of one diopter (D) in the K maximum (Kmax), or a decrease in corneal thickness at its thinnest point of 2% in a period of 6 months or that were at risk of progression [under 16-years-old with central cone defects and decrease in the corrected VA (4)] were included in the CXL group. The patients that did not tolerate contact lenses or that suffered difficulties in adapting to them, or those with greater visual demands than those obtained by optical correction were included in the ICRS implantation group. The demographic and clinical data collected included gender, age, patient’s ocular history, medical history (allergy and eye rubbing), and topical and systemic treatments.

### Ophthalmological examination

The basal pre-operative and post-operative exploration at 18 months included the uncorrected distance visual acuity (UDVA) and the corrected distance visual acuity (CDVA) as the logarithm of the minimum angle of resolution, the determination of the spherical equivalent (SE) with an autorefractometer and the exploration of the anterior segment by slit lamp biomicroscopy. A Pentacam^®^ (Pentacam HR; Oculus Optikgerate GmbH, Wetzlar, Germany) apparatus was used for the tomography study of the cornea, through which the following study variables were registered: flat keratometry (K1), steep keratometry (K2), K max, mean keratometry (Mean-K), and the minimal corneal thickness (MCT).

To assess the ocular surface variables and tear function, complementary tests were performed such as the measurement of tear osmolarity (OSM) (TearLab Osmolarity System: Reader, TearLab Co., San Diego, CA, United States), the Ocular Surface Disease Index (OSDI) questionnaire, tear break up time (TBUT), and the Schirmer test (SCH) with anesthesia. These tests were always carried out in the same order at the consultations.

### Intracorneal ring segment surgery

All the surgical procedures were carried out as outpatient treatments under topical anesthesia (double Colircusi anesthesia: a collyrium containing 1 mg/ml of tetracaine hydrochloride and 4 mg/ml oxibuprocaine hydrochloride), and strict aseptic conditions of the eye (5% iodinated povidone) and periocular area (10% iodinated povidone). For ICRS implantation, the surgical plan envisaged the number of rings to be implanted, the position of the incisions, the thickness, and the arc of the ring and the diameter of the optic zone in each case. During the intervention, the central point of the cornea was first marked and the eyeball was fixed through a vacuum system, to which the laser interface was coupled. The corneal tunnel is configured previously to work at a depth of 70–80% of the thinnest point in the rings’ trajectory. After making an opening with the femtosecond laser (Technolas, Bausch and Lomb (B&L), Munich, Germany), the rings were introduced with the aid of a Ferrara spatula at the predetermined position. After surgery, post-operative treatment with Tobradex eye drops^®^ (1 mg/ml Dexamethasone and 3 mg/ml Tobramycin) was recommended with a schedule diminishing over 1 month.

### Crosslinking

For the CXL procedure, corneal desepitheliazation was performed at 9 mm from the central diameter with 20% alcohol and riboflavin (0.1%: VibeX RapidTM, Avedro; Waltham, MA, United States) was applied every 2 min over 10 min. Subsequently, the LED lamp (Avedro^®^ KXL; Waltham, MA, United States) was put in place to apply the UVA radiation with on/off pulses over 8 min, administering a total irradiation of 7.2 J/cm^2^. Finally, the eye was cleaned with abundant physiological serum and a drop Tobradex^®^ was applied, thereafter placing a therapeutic contact lens until re-epithelialization was completed. These patients were recommended to follow the same post-operative treatment as the patients subjected to ICRS.

### Tear sample collection

All the tear samples were collected using calibrated 10 μl glass microcapillary tubes (BLAUBRAND intraMark, Wertheim, Germany). Tear samples were obtained from the inferior temporal tear meniscus, minimizing any irritation of the ocular surface or lid margin, and without the installation of anesthesia. The tear samples were collected from both eyes of each participant and immediately placed in precooled Eppendorf tubes. After tear collection, the samples were stored at −80°C in the Basque Biobank^1^ following standard operation procedures with appropriate approval of the Ethical and Scientific Committees until their analyses.

### Proteomics analyses

The proteomics analyses were carried out at the CIC bioGUNE Proteomics Platform (Derio, Bizkaia, Spain), using the Filter Aided Sample Preparation (FASP) protocol for sample processing and digestion with minor variations (45). Briefly, samples were solubilized in a buffer containing 7M Urea 2M Thiourea and 4% CHAPS and submitted to buffer exchange steps using 30 KDa cutoff AMICON filters, as described in the protocol described by Wiśniewski et al. (45). Trypsin was added at a trypsin:protein ratio of 1:50, and the mixture was incubated overnight at 37°C, dried in a RVC2 25 Speedvac concentrator (Christ) and resuspended in 0.1% Formic Acid (FA). The peptides obtained were desalted and resuspended in 0.1% FA using C18 stage tips (Millipore, St. Louis, MO, United States).

Samples (4 biological replicates except for the control condition, where 5 replicates were used) were analyzed in a novel hybrid trapped ion mobility quadrupole time of flight mass spectrometer (timsTOF Pro with PASEF: Bruker Daltonics, Bremen, Germany), coupled online to a nanoElute liquid chromatograph (Bruker, Coventry, United Kingdom). This mass spectrometer takes advantage of a novel scan mode, termed parallel accumulation serial fragmentation (PASEF), which multiplies the sequencing speed without any loss of sensitivity, and it has been proven to provide outstanding analytical speed and sensitivity for proteomics analyses. Samples (200 ng) were loaded directly onto a 15 cm Bruker nanoelute FIFTEEN C18 analytical column (Bruker) and resolved at 400 nl/min. Mass spectrometer was operated in DDA PASEF mode using the standard method provided by the manufacturer. A 30 min linear gradient (3–40% acetonitrile) was used to resolve and analyze the samples. The column was heated to 50°C in an oven.

Protein identification and quantification was carried out using the PEAKS software (Bioinformatics Solutions, Waterloo, ON, Canada). Searches against a database of canonical human Uniprot/Swissprot entries (2020_03 release, 20368 entries, no isoforms considered), with precursor and fragment tolerances of 20 ppm and 0.05 Da. Area-based label-free protein quantification was performed using the PEAKS Q module available in the PEAKS software. Only proteins identified with at least two peptides at a False Discovery Rate (FDR) < 1% at peptide level and present in at least 70% of the samples from one of the experimental groups analyzed were considered for further analysis. The data was loaded onto the Perseus platform and further processed (log2 transformation, imputation) before the application of a Student’s *t*-test for differential protein expression analysis.

The mass spectrometry proteomics data have been deposited to the ProteomeXchange Consortium *via* the PRIDE (46) partner repository with the dataset identifier PXD035655 and 10.6019/PXD035655.

### Statistical analyses

A descriptive analysis of the variables by different groups was performed using absolute and relative frequencies in the case of categorical variables, and the median and interquartile range (IQR) in the case of continuous variables. The non-parametric Mann–Whitney test was used to compare the medians of the groups.

The intensity of the spectrometry signals was transformed to the binary logarithm to reduce the effect of the variability in the results and the subsequent normalization is achieved by iterative rank-order normalization (IRON). The data obtained by mass spectrometry was compared to the human protein database (Homo sapiens database) for identification. The normal distribution of the resulting samples was assessed through a Shapiro–Wilk test and the statistical significance of the mean differences was measured using a Student *t*-test. The pooled comparison of the three groups were established by ANOVA analysis. The *p*-values calculated determine the probability that the association between the proteins in the dataset and a given canonical pathway, functional network or upstream regulator is explained by chance alone, based on a Fisher’s exact test with a *p*-value < 0.05 considered to be significant. We also produced volcano plots to identify the differences between the groups in terms of their protein composition, with a 2-fold change and α = 0.05 obtained in the test *t*-Student using a Benjamini-Hochberg FDR of 5% as the correction for the multiple tests. The differences in protein expression in each group were determined by calculating the ratios of protein expression per group. Levels of expression between 0.5 and 2-fold were considered similar. The identification of the proteins with the greatest differences between the groups (the most strongly over- or under-expressed) were established by calculating the Euclidean distance. All the data was analyzed using the R-Statistics programming software.

## Results

### Patients and clinical parameters

#### Control group

The clinical study of the Ctrl group was performed at only one-time point. This group consisted of four patients with a median age of 33 years (IQR 13.25), with no known ophthalmological or systemic pathologies of interest, nor were they receiving any topical or systemic treatments. Their visual function, refractive and topographic values, and those in reference to the eye surface and tear function were recorded (see Table 1).

**TABLE 1:** Comparison of results after treatment with baseline values.

	Ctrl	ICRS			CXL		
Variable	Baseline	Baseline	18 month	*P*-value	Baseline	18 month	*P*-value
UDVA (logMAR)	−0.04 (IRQ: 0.29)	0.73 (IQR: 0.30)	0.60 (IQR: 0.36)	0.486	0.55 (IQR: 0.27)	0.61 (IQR: 0.16)	1.000
CDVA (logMAR)	−0.13 (IRQ: 0.10)	0.22 (IQR: 0.21)	0.10 (IQR: 0.07)	0.234	0.28 (IQR: 0.17)	0.12 (IQR: 0.07)	0.309
SE (D)	0.32 (IRQ: 0.65)	−4.31 (IQR: 2.32)	−3.25 (IQR: 2.75)	0.800	−7.75 (IQR: 4.00)	−6.77 (IQR: 3.60)	0.800
K1 (D)	42.35 (IRQ: 0.93)	47.55 (IQR: 3.25)	45.95 (IQR: 2.47)	0.486	46.80 (IQR: 1.85)	46.75 (IQR: 2.60)	0.686
K2 (D)	43.25 (IRQ: 0.88)	54.50 (IQR: 7.10)	49.85 (IQR: 6.92)	0.343	51.50 (IQR: 1.90)	50.85 (IQR: 1.25)	0.886
Kmax (D)	43.60 (IRQ: 0.95)	62.00 (IQR: 6.55)	58.00 (IQR: 2.27)	0.200	61.40 (IQR: 6.20)	62.05 (IQR: 9.05)	1.000
Mean-K (D)	42.80 (IRQ: 0.83)	53.45 (IQR: 3.25)	47.65 (IQR: 4.93)	0.110	48.35 (IQR: 1.20)	48.50 (IQR: 1.30)	0.657
MCT (μ)	558.00 (IRQ: 58.25)	429.50 (IQR: 6.00)	435.50 (IQR: 6.25)	0.486	451.00 (IQR: 24.00)	436.00 (IQR: 40.00)	0.886
OSM (mOsm/L)	275.50 (IRQ: 3.50)	304.00 (IQR: 0.00)	290.50 (IQR: 9.25)	0.100	304.50 (IQR: 9.75)	313.50 (IQR: 8.50)	0.057
OSDI	2.00 (IRQ: 1.00)	6.50 (IQR: 10.0)	1.00 (IQR: 3.25)	0.301	18.00 (IQR: 12.50)	16.50 (IQR: 13.75)	0.772
TBUT (sec)	11.50 (IRQ: 3.75)	13.50 (IQR: 5.50)	16.00 (IQR: 2.50)	0.306	10.00 (IQR: 2.50)	13.50 (IQR: 7.50)	0.661
SCH (mm)	13.50 (IRQ: 4.00)	14.50 (IQR: 2.75)	10.50 (IQR: 8.25)	0.561	12.00 (IQR: 16.00)	10.50 (IQR: 15.25)	0.663

Values are expressed as median and interquartile range (IQR).

UDVA, uncorrected distance visual acuity (logMAR); CDVA, corrected distance visual acuity (logMAR); SE, spherical equivalent (diopters); K1, flat keratometry (diopters); K2, steep keratometry (diopters); Kmax, maximum keratometry (diopters); Mean-K, mean keratometry (diopters); MCT, minimal corneal thickness (μ); OSM, tear osmolarity (mOsm/L); OSDI, ocular surface disease index; TBUT, tear break up time (seconds); SCH, Schirmer’s test (mm). *P* < 0.05 show statistically significant differences.

#### The intracorneal ring segment group

The median of age of this group was of 37.5 years and 75% of these patients suffered from allergic disease such as atopic dermatitis, asthma, or allergic conjunctivitis, but unlike the CXL group only 25% of the patients indicated having or having had a habit of rubbing their eyes. Moreover, 25% of them used contact lenses. In the pre-surgical baseline condition, 50% of the eyes had moderate KC (K2 45-52D) and the rest had severe KC (K2 > 52D). Only 25% of the patients had chronic treatment with oral iron. The rest had no oral or topical prescription. In the post-operative study of these patients, a functional and refractive improvement from 0.73 to 0.60 for logMAR UCVA and from 0.22 to 0.10 for logMAR BCVA was detected. Moreover, the SE fell by 1,06D. After 18 months, the topographic values indicated a medium flattening of the cornea by 1.6D in K1, 4.65D in K2, 4D in Kmax, and 5.8D in Kmean. The increase in corneal thickness at its thinnest point was 6 microns post-operatively. The complementary ocular surface evaluation tests, OSM, TBUT, SCH, and OSDI questionnaire remained stable over time and with normal values. The differences detected were not statistically significant (Table 1).

#### Crosslinking group

The group of patients subjected to CXL had a median of age of 17.5 years and all these patients had allergic condition. Although all these patients reported chronic eye rubbing only 50% of them used topical anti-histamines. In addition, 50% of the patient were being treated with topical (inhaled) extraocular corticosteroids and 25% with systemic leukotrien receptor antagonist. Also, half of these patients were occasional users of contact lenses. In the pre-surgical baseline condition, 75% of the eyes showed moderate KC (K2 45-52D) and the rest severe KC (K2 > 52D).

After 18 months, functional and refractive outcomes demonstrated non-significant changes; worsening from 0.55 to 0.61 in UDVA logMAR, improvement from 0.28 to 0.12 in CDVA logMAR, and a reduction of 1D SE (from −7.75 to −6.77). As for the topographic values analyzed (K1, K2, Kmax, K-mean), there was less than 0.75D variability in each of these parameters and the median of MCT decreased by 15 microns after surgery. As for the complementary tests used to assess changes in ocular surface and tear function, there were no significant changes for either of these variables. However, there was a change in OSM which increased to pathological values (to mild dry eye). None of the changes recorded in the post-operative studies of this group of patients were statistically significant (Table 1).

### The nano-liquid chromatography-mass spectrometry data

A total of 447 tear proteins were identified in the samples analyzed here, in agreement with previous tear proteomic studies (21, 23). Several group-specific alterations of the tear proteome were evident in KC patients relative to the controls and proteins with different abundances were detected in all the groups studied. Comparative analyses were performed between the basal state of the three groups, Ctrl versus ICRS, Ctrl versus CXL, and ICRS versus CXL. Moreover, each group was analyzed separately at baseline and 18 months after treatment.

**FIGURE 1:**
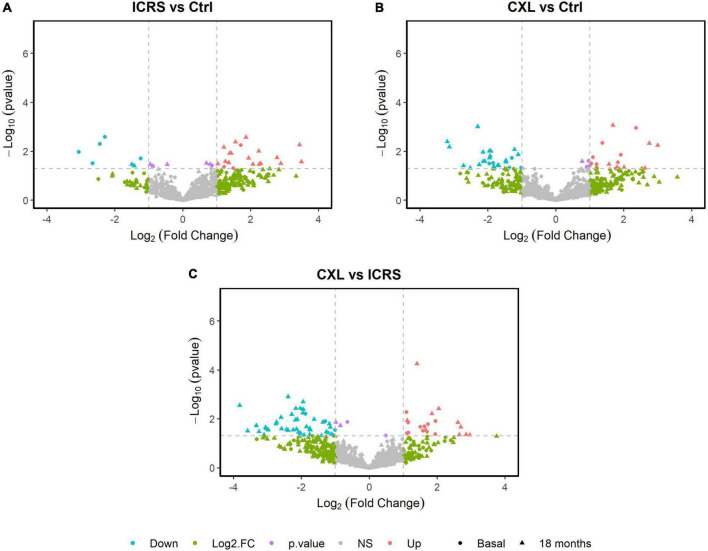
Volcano map of protein expression was performed for the pairwise comparison of the three groups; ICRS vs. Ctrl **(A)**, CXL vs. Ctrl **(B)**, and CXL vs. ICRS **(C)**, differentiating between the two time periods considered (Baseline and 18 months).

Volcano maps of gene expression were obtained to compare the three groups of patients (ICRS vs. Ctrl, CXL vs. Ctrl, and CXL vs. ICRS), distinguishing between the two time periods considered (baseline and 18 months after the intervention, Figure 1). When the two surgical techniques were compared (CXL vs. ICRS, Figure 1C), differences in protein expression between these techniques were represented at each of the two time points but not a comparison for the two time points for each group (CXL and ICRS). The proteins that displayed the greatest differences in expression (4-fold overexpression and 4-fold under-expression) when comparing the two surgical techniques (ICRS vs. Ctrl, CXL vs. Ctrl, and CXL vs. ICRS) at the baseline and after 18 months are reflected in Table 2. The most relevant selection of the proteins was established through their position in the volcano plots, ordering them according to the differences in the Euclidean distance calculated between their origin and the distance represented by the Fold change [log2 (Fold)] and the *p*-value [−log10 (*p*-value)]. The proteins with the largest distances represented those with the strongest changes in expression between the groups compared.

**TABLE 2:** Changes between surgical techniques and control group by time period and expression type.

Period	Group	Entry name	Description	Fold	*P*-value
Basal	ICRS/Ctrl	ASC-1	Activating signal cointegrator 1 complex subunit 3	3.26	0.006
		KV118	Ig kappa chain V-I region WEA	2.81	0.048
		DSP	Desmoplakin	2.32	0.043
		CALR	Calreticulin	0.20	0.003
		AT1A4	Sodium/potassium-transporting ATPase subunit alpha-4	0.18	0.005
		PROL-4	Proline-rich protein 4	0.16	0.030
		APOBEC-3A	DNA dC → dU-editing enzyme APOBEC 3A	0.12	0.011
	CXL/Ctrl	DSP	Desmoplakin	5.13	0.001
		PRG2	Bone marrow proteoglycan	3.77	0.014
		ALS	Insulin-like growth factor-binding protein complex acid	3.55	0.028
		ABCA1	ATP-binding cassette sub-family A member 1	2.58	0.005
		AMY1	Alpha-amylase 1	0.41	0.019
		PROM1	Prominin-1	0.39	0.047
		GLU2B	Glucosidase 2 subunit beta	0.28	0.030
		MUC5B	Mucin-5B	0.26	0.031
	CXL/ICRS	AKP13	A-kinase anchor protein 13	26.98	0.019
		ALS	Insulin-like growth factor-binding protein complex acid	3.87	0.012
		CATD	Cathepsin D	3.31	0.017
		PEDF	Pigment epithelium-derived factor	2.13	0.005
		ASCC3	Activating signal cointegrator 1 complex subunit 3	0.43	0.037
		CALU	Calumenin	0.41	0.013
		OLFM4	Olfactomedin-4	0.27	0.006
		GLU2B	Glucosidase 2 subunit beta	0.25	0.004
18 months	ICRS/Ctrl	CO7	Complement component C7	11.23	0.027
		MDHM	Malate dehydrogenase mitochondrial	10.83	0.005
		ASAH1	Acid ceramidase	7.38	0.031
		ELNE	Neutrophil elastase	6.83	0.018
		MUC5B	Mucin-5B	0.37	0.039
		PEDF	Pigment epithelium-derived factor	0.35	0.034
	CXL/Ctrl	CO7	Complement component C7	7.97	0.006
		HPTR	Haptoglobin-related protein	6.70	0.005
		TFF1	Trefoil factor 1	6.14	0.047
		ZA2G	Zinc-alpha-2-glycoprotein	3.19	0.001
		CAP1	Adenylyl cyclase-associated protein 1	0.20	0.001
		LV211	Ig lambda chain V-II region NIG-84	0.15	0.038
		IGHG2	Ig gamma-2 chain C region	0.11	0.007
		MUC5B	Mucin-5B	0.11	0.004
	CXL/ICRS	AKP13	A-kinase anchor protein 13	13.40	0.05
		APOE	Apolipoprotein E	7.76	0.046
		K1C16	Keratin type I cytoskeletal 16	6.09	0.014
		HPTR	Haptoglobin-related protein	4.12	0.004
		ZA2G	Zinc-alpha-2-glycoprotein	2.64	0.000
		CO1	Complement factor I	0.19	0.001
		MMP9	Matrix metalloproteinase-9	0.10	0.019
		ELNE	Neutrophil elastase	0.08	0.031
		CAP1	Adenylyl cyclase-associated protein 1	0.07	0.003

*P* < 0.05 show statistically significant differences.

**FIGURE 2:**
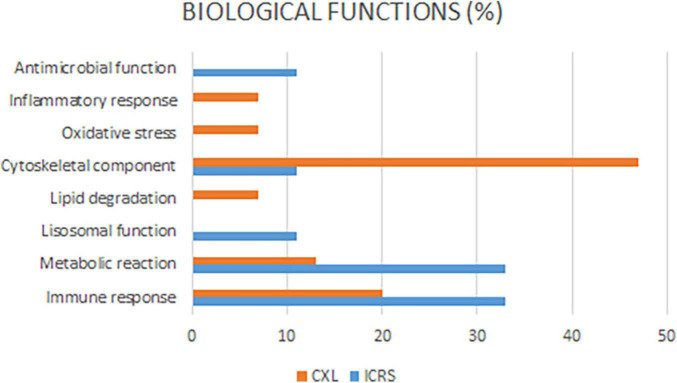
Most of the differentially expressed proteins could be localized to the extracellular and intracellular compartments once the biological context and functional annotation analyses. As a result, deregulated proteins were seen to be involved in different biological processes.

**FIGURE 3:**
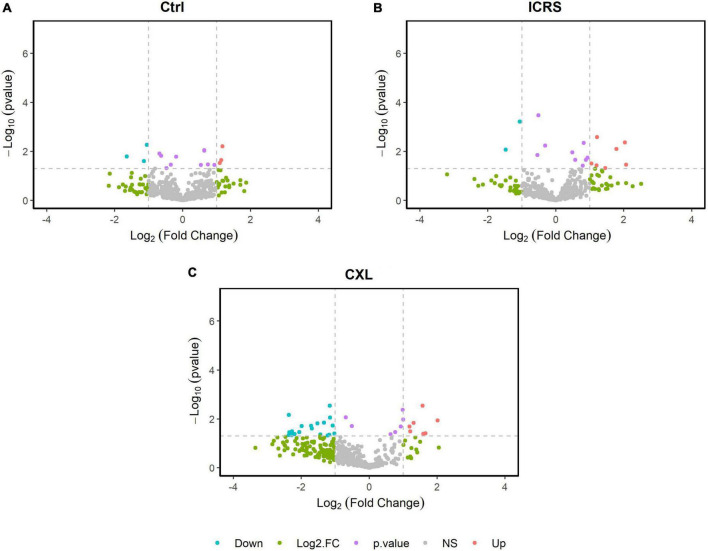
Volcano map of protein expression for the pairwise comparison of the two time periods (Baseline and 18 months), differentiating between groups; Ctrl **(A)**, ICRS **(B)**, and CXL **(C)**.

Most of the differentially expressed proteins could be localized to the extracellular and intracellular compartments once the biological context and functional annotation analyses were performed using the different gene ontology (GO) terms. As a result, deregulated proteins were seen to be involved in different biological processes. In the comparisons between the two surgical groups and the Ctrl, the biological processes that were altered in the KC patients (ICRS + CXL) at baseline were those that were deregulated as a consequence of the disease and not of the surgical intervention. Among the biological processes seen to be altered were: Immune responses (Ig kappa chain V-I region WEA), cytoskeletal components (Desmoplakin), protein synthesis (Proline-rich protein 4), metabolic reactions (Sodium/potassium-transporting ATPase subunit alpha-4, DNA dC → dU-editing enzyme) (Figure 2). On comparing the two treatment groups (ICRS and CXL), of all the processes altered the most significant was that related to collagen degradation, which was probably provoking the corneal thinning that was most pronounced in the patients subjected to CXL. This was reflected in the 26.98-fold overexpression of the protein A-kinase anchor protein 13 in the tears of the patients in the CXL group relative to that in the tear of patients subjected to ICRS. There was weaker expression of this protein after both of the surgical procedures, although it continues to be more strongly overexpressed in the CXL group than in the ICRS group 18 months after surgery (13.4-fold). The pachymetry was thinner after surgery in the CXL group than in the ICRS group. This is consistent with the corneal collagen fibbers remodeling and compaction after CXL, with a difference in corneal pachymetry between the measurement at baseline and after 18 months of −15 and +6 microns, respectively. The changes produced between the two time points (basal and 18 months’ post-surgery) were also analyzed for each of the two surgical approaches (Figure 3). At 18 months’ post-surgery, up- and downregulated proteins were detected in the two patient groups (Table 3). The changes were established separately for each of the surgical groups, allowing us to see which proteins were affected in each group of patients as a consequence of surgery. The Table 3 shows the proteins with the greatest changes in expression after each of the procedures performed.

**TABLE 3:** Proteins with the most relevant expression changes before and after the intervention by surgery type.

Group	Entry name	Description	Fold	*P*-value
ICRS	LV001	Ig lambda chain V region 4A	4.19	0.035
	HS90B	Heat shock protein HSP 90-beta	4.09	0.004
	GYLG	Glycogenin-1	3.44	0.008
	KV113	Ig kappa chain V-I region Lay	2.74	0.048
	LV301	Ig lambda chain V-III region SH	2.32	0.003
	CATD	Cathepsin D	2.30	0.038
	DESP	Desmoplakin	2.07	0.031
	LC1L1	Putative lipocalin 1-like protein 1	0.48	0.001
	AK1A1	Alcohol dehydrogenase [NADP(+)]	0.36	0.009
CXL	K1C9	Keratin type I cytoskeletal 9	4.03	0.012
	TPM2	Tropomyosin beta chain	3.15	0.039
	C1R	Complement C1r subcomponent	3.02	0.040
	ZA2G	Zinc-alpha-2-glycoprotein	2.98	0.003
	SLPI	Antileukoproteinase	2.47	0.014
	K1C10	Keratin type I cytoskeletal 10	2.30	0.032
	CY24B	Cytochrome b-245 heavy chain	2.27	0.020
	AHNK	Neuroblast differentiation-associated protein	0.45	0.003
	ML12A	Myosin regulatory light chain 12A	0.25	0.020
	F11	Protein F11	0.22	0.042
	MYH14	Myosin-14	0.21	0.033
	KV206	Ig kappa chain V-II region RPMI 6410	0.21	0.046
	SSPO	SCO-spondin	0.20	0.035
	PROF1	Profilin-1	0.19	0.045
	PGAM1	Phosphoglycerate mutase 1	0.19	0.007

*P* < 0.05 show statistically significant differences.

After ICRS (18 months) the deregulated proteins were mainly implicated in: immune responses (Ig lambda chain V region 4 A, Ig kappa chain V-I region Lay, Ig lambda chain V-III region SH), metabolic reactions {Heat shock protein HSP 90-beta, Glycogenin-1, Alcohol dehydrogenase [NADP (+)]}, antimicrobial activity (Putative lipocalin 1-like protein 1), and the cytoskeleton (Cathepsin D, Desmoplakin). By contrast, after CXL the proteins deregulated were implicated in inflammatory responses (Antileukoproteinase), oxidative stress (Cytochrome B-245 heavy chain), metabolic reactions (Phosphoglycerate mutase 1, Zinc-alpha-2-glycoprotein) and immune responses (Complement C1r sub-component, Ig kappa chain V-II region RPMI 6410), and above all they included cytoskeletal components (Keratin type I cytoskeletal 9, Tropomyosin beta chain, Keratin type I cytoskeletal 10, Neuroblast differentiation-associated protein, Myosin regulatory light chain 12A, Myosin-14, Profilin-1: Table 3).

## Discussion

The pathology of KC was classically not considered an inflammatory disease since it did not possess the typical pattern of cellular infiltrate and vascularization. However, studies in this past decade indicate that an inflammatory cascade may occur at the ocular surface of KC patients due to the presence of enzymes related to collagen degradation and corneal thinning, or through the release of pro-inflammatory cytokines or certain genetic mutations (15, 47). Elsewhere, evidence of oxidative stress was obtained through alterations to antioxidant enzymes, and the accumulation of lipid peroxidation products, elements in the nitric oxide pathways and impaired mitochondrial membrane potentials (48–51).

Here tear samples were analyzed from Ctrl subjects and KC patients subjected to two treatments, CXL or ICRS, obtained at baseline and 18 months after the intervention. From a clinical point of view, the presurgical features of these patients differ. The CXL patients are generally younger and with a greater risk of disease progression, while the ICRS patients are older and with KC that has evolved over a longer period, and that is more stable and severe (e.g., median K2 51.50D CXL, 54.40D ICRS). In addition, the need for treatment for allergic disease is higher in CXL group than in ICRS group, probably because of being a younger group, as part of asthmatic and atopic dermatitis patients show improvement of symptoms in early adulthood (52, 53). Despite these differences, the characteristics of each group did not change throughout the study period, which leads us to believe that the changes observed in patients tear proteomics may be secondary to the procedures performed in each group.

The study of the clinical parameters did not show statistically significant differences between the two study moments. The main explanation for this lack of significance could be the sample size we have (*n* = 4 in each group), the major limitation of the study. Variables related to ocular surface status did not change significantly at the 18-month post-operative period. Therefore, it seems that the changes observed in the tear proteome correspond to the effects of the surgery itself on the cornea and not to changes induced by the procedures on the ocular surface.

Intracorneal ring segment is an effective technique to regulate the cornea that can be explained by the Barraquer thickness law, whereby removing tissue from the center of the cornea or adding tissue to the periphery flattens the curvature of the cornea. This effect is directly proportional to the thickness and inversely proportional to the ring diameter (54). Ferrara rings are made of biocompatible and inert poly (methyl methacrylate). Prior to their implantation in humans their biocompatibility was demonstrated in rabbit corneas, with good medium term tolerance and maintaining the transparency of the central cornea (55). However, the corneal response to this biomaterial is still under study and as yet, aspects such as the importance of the changes in protein expression in the cornea following ICRS and how this may affect the stability of the refractive and visual consequences, as well as in any post-operative complications remains unclear.

For introduction of the Ferrara rings, it is necessary to make of intrastromal tunnel manually or using a femtosecond laser. This laser employs thousands of very short pulses of light close to the infrared spectrum (1053 nm) to create microcavities that separate the corneal tissue (56). As such, both the creation of the intrastromal tunnels with the femtosecond laser as well as the implantation of the rings could induce structural and biomechanical changes, on top of the biological changes in the cornea, which could be reflected in the alterations to the tear proteome of these patients. To better understand the biological processes initiated by this surgery and that develop as a consequence of these procedures, the basal proteome of the tear was compared with that at 18 months’ post-surgery of patients subjected to ICRS.

At baseline, proteomic differences were evident in these patients relative to the Ctrl, with the overexpression of some proteins and the downregulation of others. At 18 months’ post-surgery, significant differences relative to the baseline were seen for 9 proteins, 7 of which were overexpressed, and 2 downregulated. Among the overexpressed proteins were desmoplakin and cathepsin D, both of which are related to desmosomes. These cell structures are fundamental for intercellular adhesion and the adhesion between cells and the cytoskeleton, as well as in the resistance of the epithelium to mechanical stress (57). However, these proteins have opposing roles, whereby desmoplakin is a desmosome protein that couples intermediate filaments (IFs) to the desmosome plaque (58), while cathepsin D is a protease that acts on fibronectin and laminin in the extracellular matrix (ECM), the expression of which in the desmosomes potentially producing cell shedding (59).

The overexpression of both these proteins could be explained by the findings *in vivo* and *ex vivo* from corneas subjected to ICRS. Confocal microscopy identified large hyperreflective nuclei in the transition from the basal epithelial layers to the intermediate layers in the region covering the rings of some such corneas, as well as signs of an increase in epithelial mitosis (60). The increase in desmoplakin expression could correspond to the stimulation of epithelial mitosis. Moreover, epithelial hypoplasia in the epithelium covering the rings has been observed, whilst the central epithelium retains a normal structure (61, 62). The epithelium between these two zones is hypertrophic (62), which is related to the biological stress produced by the implant (60). In fact, on removing the rings a recovery of the epithelium is observed (61). The increased cathepsin D expression could be related to this hypoplasia, given that it favors cell shedding.

In addition to epithelial changes, an increase in cell density has been described in the stroma adjacent to the rings (52, 55). However, a reduction in CD34^+^ cells has also been observed in the stroma immediately above and below the rings, which might be related to a reduction in the number of keratocytes, as well as to the changes produced in their phenotype, probably toward phenotypes generating collagen (61). Likewise, anomalous ECM components have been detected around the intrastromal rings that are not evident in the healthy cornea but that appear during repair processes. These components include tenascin-C, fibrilin-1, and proteinases specific to collagen type III, IV (a1/a2), and XIV. In particular stromelysins and some cathepsin F and H have been detected (63). It has been proposed that this overexpression may be due to the participation of keratocytes in stromal remodeling and in degrading the excess fibrosis in the ECM that surrounds the rings as part of the reparative events observed after ring implantation (63). The over expression of cathepsin D in our patients could also form part of this remodeling process. Other members of the cathepsin family have been seen to be elevated in the tear and cornea of patients with KC, like cathepsin S (CATS), relating the amounts in the tear to the increase in corneal curvature (64). Cathepsin B, G and F are also related to secondary fibrosis in ruptures of the Bowman membrane (64), although these other cathepsins were not seen to be overexpressed in our patients.

Both in the pre-operative and post-operative analysis, overexpression of some Immunoglobulin (Ig) chains was seen in the ICRS patients. In the post-operative period, there was a significant increase in the basal expression of Ig chains lambda V region 4A, Ig kappa V-I region Lay, and Ig lambda chain V-III region SH. These chains constitute the light chains of the IgG, IgA, IgM, IgD, and IgE isotypes (65). IgA is the principal Ig in the tear and it has been seen to be downregulated in patients with KC, reinforcing the inflammatory and immunological aspects of the pathology (66). In terms of the light and heavy Ig chains, some differences in their expression have been reported in patients with KC (65, 67), although the significance of this deregulation is unclear. The atopic condition of these patients must be borne in mind given the distinct immunological profile of these patients relative to the control subjects.

The other treatment used, CXL prevents KC by increasing the covalent bonding between the collagen fibers of the corneal stroma, thereby improving the mechanical resistance of the cornea to deformation (68). Several studies have shown that corneal collagen CXL can delay or prevent the progression of KC, and prevent post-operative corneal dilation (69, 70). After CXL, the diameter of the collagen fibers in the anterior matrix of the cornea increases and there is a loss of keratocytes in the treated area. It might be speculated that the change in corneal hardness after CXL could be due to the differential expression of proteins present in the cornea.

To understand the biological changes in the cornea during corneal remodeling after CXL, we studied the changes in protein expression in the tear of patients before and 18 months after surgery, identifying significant differences in the expression of certain proteins. We focused on proteins that exhibited a tendency to change their expression over time. As indicated previously (67), there were more deregulated proteins at baseline relative to the Ctrl, probably due to an increase in the proteases present in KC and a decrease in keratocyte secretion. However, 18 months after CXL more overexpressed proteins appeared than at baseline, probably due to the changes that occur as a result of the treatment.

Many of the proteins that appear to be overexpressed at 18 months after CXL surgery were components of the cytoskeleton, with Keratin type I cytoskeletal 9 (4.03-fold) and Tropomyosin beta chain (3.15-fold) those most strongly expressed. Keratins are proteins that form the cytoskeleton of epithelial cells, and changes in keratin expression have contributed to the evolutionary adaptation of epithelia to different environments (71). The keratins present in the corneal epithelium and other proteins related to the cytoskeleton could be altered by the effect of UV light on the riboflavin used in the surgical procedure. A significant decrease in collagen types I, III, V, and XII, as well as in the lumican proteins has been proposed in keratoconic corneas (72), as has a reduction in the interfibrillar distance of collagen lamellae and an increase in proteoglycans with abnormalities in their configuration as the disease progresses (73). However, the increase in collagen-related proteins and cytoskeleton components after CXL reflects an active process affecting collagen fibers, strengthening these even though corneal thinning was not stabilized as it is in the ICRS patients. In the patients with more active and aggressive KC who were to undergo CXL, Insulin-like growth factor (IGF) was overexpressed at baseline in the tear. As reported previously when the *ex vivo* modulation of the healing process was studied in keratoconic corneas, more fibroblast growth factor 2 (FGF-2), platelet-derived growth factor (PDGF) and epidermal growth factor (EGF) was found in keratoconic corneas than in the controls, although secondary injury *ex vivo* reduced the EGF, FGF-2, and PDGF concentrations to undetectable levels (74, 75). Accordingly, it was proposed that dysregulation of repair pathways in KC causes the cornea to appear in a state of perpetual injury, even though some repair responses to secondary injuries such as rubbing or contact lens wear are shown.

Here, IGF does not appear in the tears of patients with KC at 18 months in either the CXL or ICRS groups, concluding that the treatments provoked an aggression that caused a decrease in its initial concentration. In addition, proteins related to inflammation were overexpressed, like the antileukoproteinase inhibitor (SLPI, 2.47-fold), and Cytochrome b-245 heavy chain (CY24B, 2.27-fold) related to oxidative stress, indicating that 18 months after treatment, active processes may persist in the corneal microenvironment. The inflammatory process has already been reported and either corneal rubbing of the eyelid, the use of contact lenses or other secondary reactions occurring in the cornea produce markers of inflammation to appear in the tear of patients with KC (47, 64, 76–78). Here, the SLPI protein was seen to be overexpressed, probably due to the intracellular activity caused by treatment. By contrast, the metalloproteinase 9 (MMP-9) that has been widely reported in KC is significantly downregulated (0.10-fold) 18 months after CXL. The decrease in MMP-9 expression at 18 months was greater in CXL patients than in ICRS patients, which may be due to the effect of covalent bonding between collagen fibers that strengthens their resistance and prevents the action of metalloproteases that degrade collagen.

Cells respond to environmental signals by mobilizing signal transduction cascades involving protein kinases and phosphatases. The correct organization of these enzymes in space and time drives the efficient and precise transmission of chemical signals. Cyclic AMP-dependent protein kinase A is compartmentalized through its association with the AKAPs, a family of scaffolds that constrain signaling enzymes to drive essential physiological events. Recently, it was recognized that defective signaling in certain endocrine disorders and cancers proceeds through pathological AKAP complexes (79). Among these proteins, AKAP4 and AKAP9 have been extensively studied as cancer-promoting factors, whereas AKAP12 and recently AKAP13 have been shown to play the opposite role, although their mechanism of action has not been studied in depth. After 18 months of CXL treatment patients still had a corneal thinning of 15 microns compared to the baseline. This may be explained by the strong tear overexpression of the protein A-kinase anchor protein 13 (AKAP13) at baseline and post-surgery, up to 26- and 13-fold, respectively, and it was more strongly expressed in CXL patients whose thinning is more active than in patients with a more stable KC that underwent ICRS.

The cornea is a collagen-rich tissue whose thickness is closely related to normal vision. In a metanalysis on corneal thinning 16 new loci were identified in more than 20,000 European and Asian individuals, some of which conferred a relatively high risk for KC, highlighting the possible involvement of genes associated with the pathogenesis of this disease (80). Functional annotations prioritized eight genes harboring SNPs with strong evidence of regulatory potential (ADAMSTS6, ARID5B, FOXO1, AKAP13, COL4A3, COL8A2, TBL1XR1, and KCMB2). The genes associated with corneal thinning were also shown to be implicated in pathways related to collagen physiology and the KC phenotype, and some of them were implicated in an interaction network involving both (80). Further studies on AKAP13 in KC patients are needed, as its strong overexpression even after treatment (13-fold) makes us suspect that it could be a good tear biomarker for KC patients with strong collagen degradation and corneal thinning.

In conclusion, the study performed here demonstrates the changes in the tear protein profile of KC patients 18 months after two surgical treatments, ICRS and CXL. The changes observed are probably due to the treatments performed and to the pathology, and not so much to the changes produced by the treatments on the ocular surface. Indeed, at 18 months after surgery there were no alterations to the variables used to assess the eye surface. Among the deregulated proteins detected, AKAP-13 deserves special attention because of its involvement in corneal thinning, and due to its strong overexpression in the tears of patients with more active KC and with a more rapid disease progression. However, it should be noted that the results obtained here cannot be directly extrapolated as this was a pilot study performed on a small number of patients, although they should serve as the basis for future studies on larger populations to see if these results are reproducible.

## Data availability statement

The mass spectrometry proteomics data have been deposited to the ProteomeXchange Consortium *via* the PRIDE (46) partner repository with the dataset identifier: 10.6019/PXD035655.

## Ethics statement

The studies involving human participants were reviewed and approved by Ethics Committee at the Hospital Universitario Donostia (Code: 2015120). Written informed consent to participate in this study was provided by the participants’ legal guardian/next of kin.

## Author contributions

AA contributed to conception and design of the study, contributed to analysis and interpretation of the data, and drafted the manuscript. All authors contributed to acquisition of data, revised the manuscript critically for important intellectual content, read and approved the final version of this manuscript, and agreed to be accountable for all aspects of the work in terms of the accuracy or integrity of any part of the work.

## Footnotes

1
www.biobancovasco.org
